# Adverse effects of the PENTO(CLO) protocol in the prevention and management of iatrogenic head and neck bone necrosis in cancer patients: A systematic review and meta-analysis

**DOI:** 10.1007/s00520-026-10428-0

**Published:** 2026-02-20

**Authors:** Marco Tulio Leandro Ribeiro, Caique Mariano Pedroso, Mariana Mayume Carvalho Kadooka, Maria Eduarda Pérez-de-Oliveira, Fabio Ramoa Pires, Márcio Ajudarte Lopes, Alan Roger Santos-Silva

**Affiliations:** 1 Department of Oral Diagnosis, Piracicaba Dental School, University of Campinas, Av. Limeira, 901 – CEP, Piracicaba, 13414-903 Brazil; 2 Department of Clinical and Preventive Dentistry, Center for Health Sciences, Federal University of Pernambuco, Recife, Brazil; 3 Oral Pathology, Department of Diagnosis and Therapeutics, Dental School, Rio de Janeiro State University, Rio de Janeiro, Brazil; 4 Postgraduate Program in Dentistry, Estácio de Sá University, Rio de Janeiro, Brazil

**Keywords:** Osteoradionecrosis, Osteonecrosis of the jaws, Pentoxifylline, Tocopherols, Clodronic acid, Adverse effects

## Abstract

**Purpose:**

To assess the proportion of adverse effects (AEs) associated with the use of PENTO or PENTOCLO protocols for the prevention and management of osteoradionecrosis (ORN) and medication-related osteonecrosis of the jaw (MRONJ).

**Methods:**

A systematic literature search was conducted across six databases (PubMed, Scopus, Embase, Web of Science, LILACS, and Cochrane Library) and gray literature, with no restrictions on date or language. Studies were eligible if they involved adults (≥ 18 years) with or at risk for ORN or MRONJ and reported AEs associated with PENTO or PENTOCLO for prevention or treatment. A proportion meta-analysis estimated the overall frequency of AEs. Subgroup analyses compared AE rates between prevention and treatment contexts and between the two regimens.

**Results:**

Of 1,075 records screened, 9 studies met the inclusion criteria. No studies reported AEs in MRONJ patients; all focused on ORN. The pooled AE proportion was 15% (95% CI: 3.6%–11.5%; *p* < 0.1; I^2^ = 55.8%). Gastrointestinal symptoms were the most reported AEs (46.38%), followed by neurovegetative effects (18.84%). AEs were more frequent in treatment settings and more prevalent in patients using PENTOCLO (28%).

**Conclusion:**

The PENTO(CLO) protocols were associated with a 15% overall AE rate, predominantly gastrointestinal symptoms. AEs occurred more often during treatment and with the PENTOCLO regimen. These findings highlight the need for close monitoring and further studies to assess safety in MRONJ patients.

**Supplementary information:**

The online version contains supplementary material available at 10.1007/s00520-026-10428-0.

## Introduction

The oncologic management of malignant neoplasms is frequently associated with a wide range of iatrogenic complications that can compromise patient’s quality of life, both during treatment and in the long term [1–3]. Among the most clinically significant side effects in the head and neck region are maxillofacial osteonecrosis, particularly osteoradionecrosis (ORN) and medication-related osteonecrosis of the jaw (MRONJ). These osteonecrosis exhibit considerable overlap in their clinical presentation, diagnostic challenges, and therapeutic approaches [4–6]. Both arise from cumulative tissue damage, whether due to exposure to ionizing radiation or the prolonged use of antiresorptive and antiangiogenic agents [7, 8].

In recent years, research has been directed toward developing novel prophylactic and therapeutic strategies to mitigate the deleterious sequelae of cancer treatment and promote biological conditions favorable to bone repair [7, 9, 10]. Nevertheless, the clinical management of maxillofacial osteonecrosis remains challenging due to its multifactorial pathogenesis, the unpredictability of therapeutic outcomes, and the limitations of conventional modalities such as empirical antibiotic therapy, hyperbaric oxygen, and invasive surgical interventions [11–14].

In this context, pharmacologically oriented strategies have gained increasing prominence for the prevention or treatment of both ORN and MRONJ, with particular emphasis on the PENTO and PENTOCLO protocols. These regimens comprise the administration of pentoxifylline (PEN) and tocopherol (TO), either alone or in combination with clodronate (CLO), and have been investigated for their potential synergistic effects in modulating radiation-induced fibrosis, reducing oxidative stress, preserving endothelial integrity, and restoring chronically impaired microvascular networks [15–17]. A growing body of evidence suggests that this therapeutic triad may enhance functional neovascularization, improve tissue perfusion, and potentially reverse persistent hypoxia in irradiated or metabolically compromised tissues [13, 18, 19].

Beyond their preliminary therapeutic efficacy, these protocols are also recognized for their ease of administration, high patient adherence, and relatively low cost compared to more invasive interventions [20]. These attributes make them particularly attractive in oncologic contexts where therapeutic options may be limited [20–22]. Despite their emerging clinical application, a critical gap persists regarding the pharmacological safety profile of these agents, especially in populations affected by immunosuppression, malnutrition, polypharmacy, or compromised hepatic and renal function [23, 24].

Reports of adverse effects (AEs) associated with PENTO(CLO) therapy remain relatively scarce. Nevertheless, a few studies reported a potentially significant systemic effects, including cardiovascular events, gastrointestinal disturbances, neurovegetative symptoms, skin reactions and hematologic abnormalities [25, 26]. The possibility of clinically relevant drug interactions and indirect immunomodulatory effects must also be considered, particularly given the fragile clinical status of many patients receiving this therapy [26, 27].

The lack of systematic AEs monitoring and the scarcity of robust longitudinal data hinder a comprehensive assessment of the risk–benefit profile of the PENTO(CLO) in clinical practice. Given their therapeutic complexity and growing use, a rigorous methodological evaluation of their potential AEs is critically warranted. This review aims to synthesize the available evidence on AEs associated with PENTO and PENTOCLO in the management of ORN and MRONJ.

## Methods

### Study design

This systematic review was conducted in alignment with the guidelines outlined in *The Cochrane Handbook for Systematic Reviews of Interventions* [28], and its reporting adhered to the *PRISMA Statement recommendations* [29] (Appendix A). The review protocol was prospectively registered in the International Prospective Register of Systematic Reviews (*PROSPERO – CRD42025624121*).

### Eligibility criteria

Eligibility criteria were defined based on the PECOS framework (Population, Exposure, Comparator, Outcome, and Study Design): (P) adult patients diagnosed with or at risk of ORN or MRONJ; (E) prevention or treatment using the PENTO(CLO) protocol; (C) not applicable; (O) occurrence of AEs associated with the protocols; and (S) clinical trials (both randomized and non-randomized) and observational studies, including cohort, cross-sectional, and case–control designs.

The inclusion criteria comprised studies involving adult participants (≥ 18 years) of either sex, who had been diagnosed with, or were considered at risk for, ORN or MRONJ and had received the PENTO or PENTOCLO protocol for either prophylactic or therapeutic purposes. To be deemed eligible, studies were required to report the occurrence of any AEs during the follow-up period related to protocol administration. No restrictions were imposed regarding publication date or language.

Exclusion criteria comprised: (1) studies with designs such as reviews, overviews, case reports, case series, research protocols, brief communications, letters to the editor, conference abstracts, and in vitro or animal studies; (2) studies involving patients younger than 18 years of age; (3) studies in which participants underwent more than one type of intervention for the prevention or management of ORN or MRONJ; and (4) articles for which the full text was unavailable, as well as clinical trials without available results or that were not yet completed.

### Information sources and search strategy

A comprehensive search was conducted on February 24, 2025, across six electronic databases: PubMed, Scopus, Embase, Web of Science, LILACS, and the Cochrane Library. Additional searches were carried out in gray literature sources including Google Scholar, ProQuest, ClinicalTrials.gov, and Ovid. Reference lists of included studies were also screened to identify potentially eligible studies that may not have been captured through the primary search strategy.

The following main key words were applied “pentoxifylline and tocopherol” or “pentoxifylline-tocopherol-clodronate” or “PENTO” or “PENTOCLO” and “Bisphosphonate Associated Osteonecrosis of the Jaw” or “Medication-Related Osteonecrosis of the Jaw” or “BRONJ” or “MRONJ” and “adverse effects” or “side effects” or “oncological safety”. The complete search strategies, including all search terms and Boolean operators used for each database, are detailed in the supplementary material Appendix B.

### Selection process

A two-stage deduplication process was performed. In the first stage, one reviewer (M.T.L.R.) exported search results to EndNote (EndNote X7, Thomson Reuters, Philadelphia, PA), where duplicates were removed. Subsequently, the remaining records were imported into the Rayyan platform (Rayyan, Qatar Computing Research Institute) and manually reviewed to eliminate any residual duplicates overlooked during the initial step.

Study selection was carried out in two phases. In the first phase, two independent reviewers (M.T.L.R. and C.M.P.) assessed the titles and abstracts. Records that did not meet the inclusion criteria were discarded. In the second phase, full-text screening was performed, and reasons for exclusion were systematically documented. Disagreements between reviewers were resolved by a third reviewer (A.R.S.S.), who provide a critical assessment and the final decision.

### Data items and collection process

Data from each included study were extracted by one reviewer (M.T.L.R.) and independently cross-validated by a second reviewer (C.M.P.) to ensure the accuracy and integrity of the extracted information. Any discrepancies between reviewers were resolved through the intervention of a third reviewer (A.R.S.S.). Extracted variables included: author, year, study design, country, study objective (prevention or treatment), PENTO(CLO) protocol regimen, adjunctive medications, duration of protocol, reported AEs and their frequency, strategies employed for AEs management, treatment discontinuation due to AEs, and evidence of long-term safety.

### Risk of bias assessment

Two reviewers (M.T.L.R. and C.M.P.) independently assessed risk of bias using the *Joanna Briggs Institute (JBI) Critical Appraisal Checklists* selected according to each study design (cohort, cross-sectional, and quasi-experimental studies) [30–32]. Each item was rated as “yes,” “no,” or “unclear.” Before initiating the process, all reviewers aligned their interpretation of each criterion to ensure consistency, and disagreements were adjudicated by a third reviewer (A.R.S.S.). To obtain an overall risk-of-bias score for each study, a weighted score was calculated for each study: “yes” were assigned a weight of 100%, “unclear” 50%, and “no” 0%. Scores were used to classify studies as high risk (≤ 49%), moderate risk (50%–69%), or low risk (≥ 70%). To visually synthesize the risk of bias assessments, the *Review Manager (RevMan) version 5.4* (Cochrane Collaboration, London, UK) was used to generate both the summary figure and the risk of bias graph.

### Effect of measures

The primary outcome was the pooled proportion of AEs occurring during the administration of the PENTO(CLO) protocol, regardless of treatment duration or clinical indication. Secondary outcomes included (1) the comparison of AE occurrence between therapeutic and preventive contexts, and (2) the comparison of AE incidence between the PENTO and PENTOCLO protocols. For the purposes of this review, an AE was defined as any undesirable or unintended clinical sign or symptom temporally associated with the use of these protocols. AE data were reported as absolute or relative frequencies and expressed with corresponding 95% confidence intervals (CIs), calculated based on the total number of reported events.

### Synthesis of results

Meta-analyses were performed using *R Studio—Version 1.4.1717* (Boston, MA) with the meta-package. Proportion meta-analyses were conducted using the inverse-variance method with a restricted maximum-likelihood estimator for between-study variance (τ^2^), to assess frequency of AEs. Heterogeneity was assessed through I^2^ and Cochran’s Q. Subgroup analyses were performed to explore heterogeneity and to observe variations in AEs proportions between treatment vs prevention and PENTO vs PENTOCLO. The random-effects model was used as it is the most appropriate model for studies conducted in different populations.

## Results

### Study selection

**Fig. 1:**
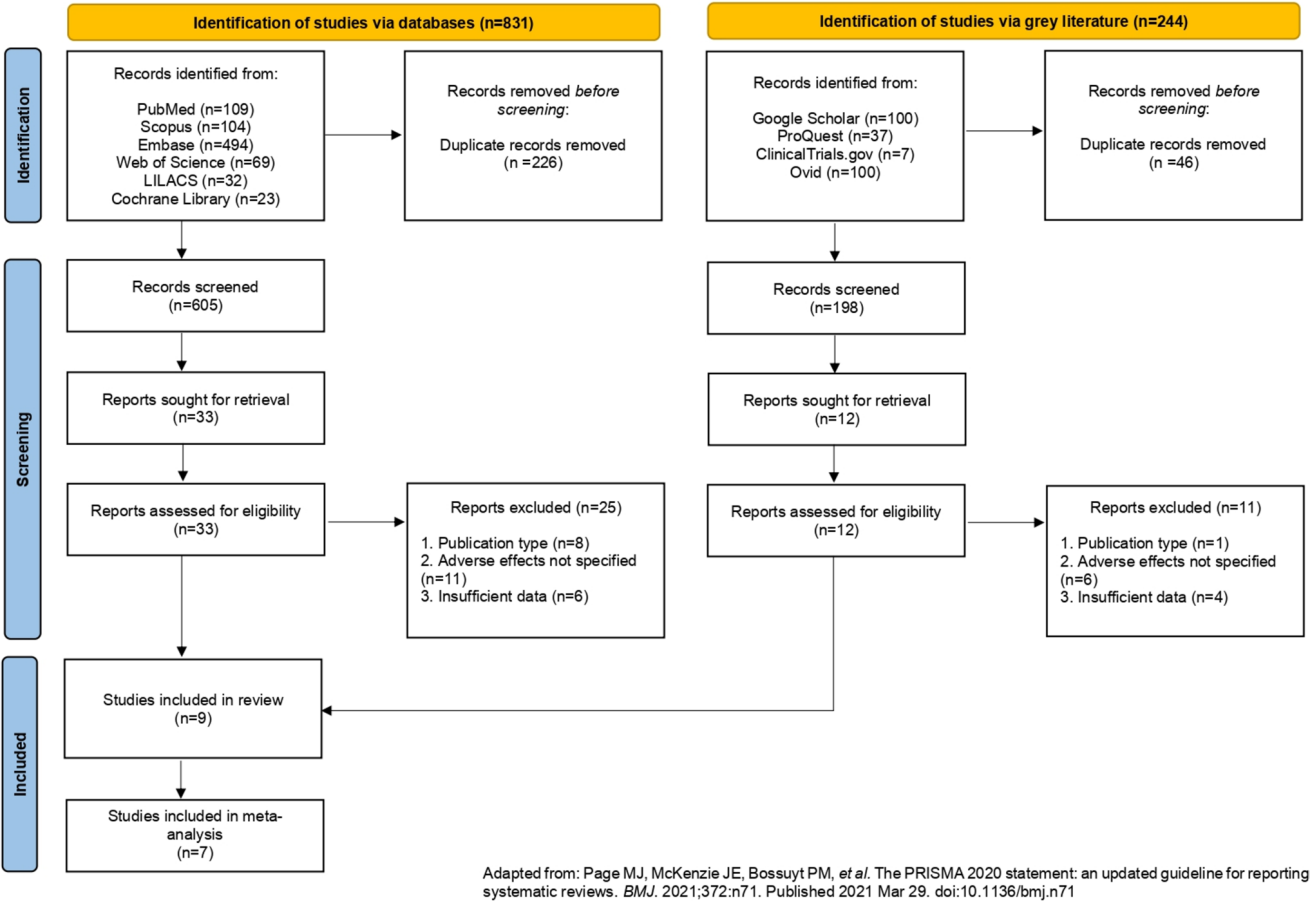
Flowchart illustrating the systematic study selection process in accordance with the PRISMA 2020 framework

The initial search across electronic databases retrieved 831 records based on the predefined search strategy. An additional 244 records were identified through gray literature sources, resulting in a total of 1,075 potentially relevant studies. After a two-stages deduplication process, a total of 803 unique records remained for screening. Titles and abstracts screening excluded 758 studies that did not meet eligibility criteria, leaving 45 articles for full-text review. Of these, only 9 met all methodological and content-related inclusion criteria and were included in the final synthesis (Fig. 1) [25, 33–40]. A comprehensive list of excluded studies, accompanied by the respective justifications for exclusion, is provided in Appendix C.

### Characteristics of included studies

AEs associated with the use of the PENTO(CLO) protocol were reported across diverse clinical contexts. Two studies focused on preventive use [33, 36], while five evaluated therapeutic applications [25, 33, 34, 37], and two encompassed both preventive and therapeutic scenarios [36, 40] (Supplementary Table 1). These studies were conducted between 2011 and 2024 in the United Kingdom, France, the United States, and India encompassed 562 patients with 69 reported AEs. All studies addressed ORN and employed either the PENTO or PENTOCLO protocol. Although specific searches were performed to identify relevant studies on MRONJ, no studies met the required methodological criteria or provided the necessary outcome data to be eligible for inclusion in this review.

Three studies evaluated the PENTO protocol for the prevention or treatment of ORN, enrolling a total of 166 participants, with sample sizes ranging from 13 to 110 patients. Across these studies, 21 AEs were reported. Four studies assessed the PENTOCLO protocol for treatment of 141 patients, with sample sizes between 27 and 54, and a total of 37 AEs recorded. Additionally, two studies simultaneously investigated both the PENTO and PENTOCLO protocols for either the prevention or management of ORN, comprising 255 patients in total, ranging from 36 to 219 per study, and reporting 11 AEs.

Among the regimens, PENTOCLO was the most frequently used. The duration of protocol ranged from 1 to 37 months. Most AEs occurred during the early phases of treatment (typically within the first 1 to 4 weeks), with a higher frequency observed in studies employing the PENTOCLO combination. Regarding supportive medications that may potentially influence the occurrence or severity of AEs, antibiotics were the most frequently used. Additional details on the general and specific characteristics of the included studies are presented in Supplementary Table 2.

### Individual results

#### AEs related to the PENTO protocol

Hayashi et al*.* (2015) treated 13 patients with established ORN and detected no AEs during therapy or follow-up [34]. Aggarwal et al*.* (2017) administered PENTO prophylactically to 110 irradiated patients undergoing dental extractions; 10 experienced nausea, headache, and gastric irritation. No data were provided on the frequency or management of these AEs [35]. Patel et al*.* (2018) evaluated both preventive and therapeutic use in 43 patients, reporting 10 AEs, including malaise, gastric irritation, nausea, hallucinations, drowsiness, nose bleeding, rash, and vomiting, mostly during the first month [36]. Most resolved within 1–4 weeks; however, six participants discontinued treatment use due to persistent intolerance. Table 1 presents the number of AEs reported in each study.

**Table 1:** AEs Reported Individually by Study

Author/Year	Sample—Patients with Adverse Effects (n)	Description of Adverse Effects						Long-Term Safety
		Type	Events (nº)	Event frequency	Duration	Management of adverse effects	Interruption due to adverse effects	
Delanian et al*.,* 2011	12	Nausea/Epigastralgia	4	Early phase of tratament	2–4 weeks	Omeprazole/Heptaminol or Reduction in PTX dosage to 400 mg/day	None of the patients discontinued treatment	No late severe adverse effects were reported
		Asthenia	2					
		Gastrostomy issues	2					
		Vertigo	1					
		Insomnia	1					
		Headache	1					
		Diarrhea	1			Reduction of CLO dosage to 800 mg/day		
Robard et al*., 2014*	6	Nausea	3	Not Reported	Not Reported	Reduction of CLO dosage	None of the patients discontinued treatment	No late severe adverse effects were reported
		Other minor effects	3					
Hayashi et al*.*, 2015	0	No adverse effects were observed	––––-	––––-	––––-	––––-	None of the patients discontinued treatment	No late adverse effects were reported
Aggarwal et al*.*, 2017	10	Nausea	Not reported individually	Not Reported	Not Reported	Not Reported	No descontinuation reported	No late adverse effects were reported
		Headache						
		Gastric irritation						
Patel et al*.*, 2018	11	Malaise	2	Intermitent use for 1 month	1–4 weeks	Discontinuation of the protocol	Six patients withdrew from the protocol	No late adverse effects were reported
		Gastric irritation	2	Early phase of treatment				
		Nausea	2					
		Hallucinations	1					
		Drowsiness	1					
		Nose bleeding	1					
		Rash	1					
		Vomiting	1					
Dissard et al*.*, 2019	15	Diarrhea	6	Not Reported	Not Reported	Reduction in the protocol dosage	No protocol interruptions due to adverse events were reported	No late adverse effects were reported
		Epigastralgia	3					
		Asthenia	3					
		Nausea	2					
		Insomnia	1					
Samani et al*.*, 2022	8	Not reported separately	––––-	Not Reported	Not Reported	Discontinuation of the protocol	Eight patients withdrew from the protocol	No late adverse effects were reported
Willcocks et al*.*, 2022	4	Rash	1	Not Reported	Not Reported	Not Reported	One patient withdrew from the protocol due to persistent nausea, gastrointestinal irritation and vomiting	No late adverse effects were reported
		Nausea	2					
		Gastrointestinal Irritation	2					
		Vomiting	1					
		Oral Burning Sensation	1			Discontinuation of CHX		
Jawad et al*.*, 2024	3	Gastrointestinal Irritation	3	Not Reported	Not Reported	Not Reported	Not Reported	No late adverse effects were reported

#### AEs of PENTOCLO protocol

Delanian et al*.* (2011) included 54 patients, of whom 12 experienced AEs, including nausea, epigastralgia, asthenia, headache, vertigo, insomnia, transient gastrostomy discomfort, and diarrhea. Management strategies involved the use of proton-pump inhibitors, supportive heptaminol, and temporary dose reduction of pentoxifylline; in cases of diarrhea, the clodronate dose was reduced. All symptoms resolved within two to four weeks [25]. Robard et al*.* reported six AEs (nausea and three unspecified minor symptoms) among 27 patients, all of which resolved following clodronate dose adjustment [33]. In the study by Dissard et al*.*, 15 of 27 patients reported AEs such as diarrhea, epigastralgia, asthenia, nausea, or insomnia. All cases were effectively managed with dose reductions, without treatment discontinuation [37].

Willcocks et al*.* observed that four patients experienced one or more AEs, including cutaneous reactions, nausea, gastrointestinal irritation, vomiting, and oral-burning sensation. One patient discontinued treatment due to persistent gastrointestinal intolerance [39]. The total number of AEs reported in each study is summarized in Table 1.

#### AEs related to the PENTO/PENTOCLO protocol

Samani et al*.*, used PENTO for prevention and PENTOCLO for treatment. Among the 219 patients monitored, eight experienced AEs; however, the study neither delineated the specific nature of these events nor their duration. All eight individuals discontinued the regimen, and no late AEs were recorded [38]. Jawad et al*.* enrolled 36 patients, implementing PENTO for prevention and PENTOCLO for treatment. Three subjects reported gastric irritation during therapy; the study did not specify the temporal course or management of these events, and no treatment discontinuations or delayed AEs were observed [40]. Table 1 presents the number of AEs reported in each study.

### Risk of bias

The risk-of-bias assessment revealed four studies at high risk, three at moderate risk, and two at low risk (Supplementary Table 3). Among the seven included cohort studies, Samani et al*.* was rated as low risk, with incomplete follow-up reporting being its only limitation [38]. Dissard et al*.* and Jawad et al*.* were judged to have moderate risk due to the absence of a comparator group, lack of clear strategies to address confounding, and incomplete follow-up data [37, 40]. Robard et al*.*, Hayashi et al*.*, Aggarwal et al*.*, and Patel et al*.* were categorized as high risk owing to imprecise exposure measurement, limited control of confounders, and inadequate statistical justification [33–36] (Supplementary Table 3; Supplementary Fig. 1).

The cross-sectional study by Willcocks et al*.* was assessed as moderate risk. Although the sampling frame and study setting were clearly described, the absence of confounder management strategies and reliance on outcome measures of uncertain validity lowered its rating [39] (Supplementary Table 3; Supplementary Fig. 2). The non-randomized clinical trial by Delanian et al*.* met most quality criteria and was classified as low risk, however, the lack of a control group and baseline comparability limited confidence in causal inference [25] (Supplementary Table 3; Supplementary Fig. 3).

### Synthesis of results

#### AEs associated with PENTO(CLO)

**Fig. 2:**
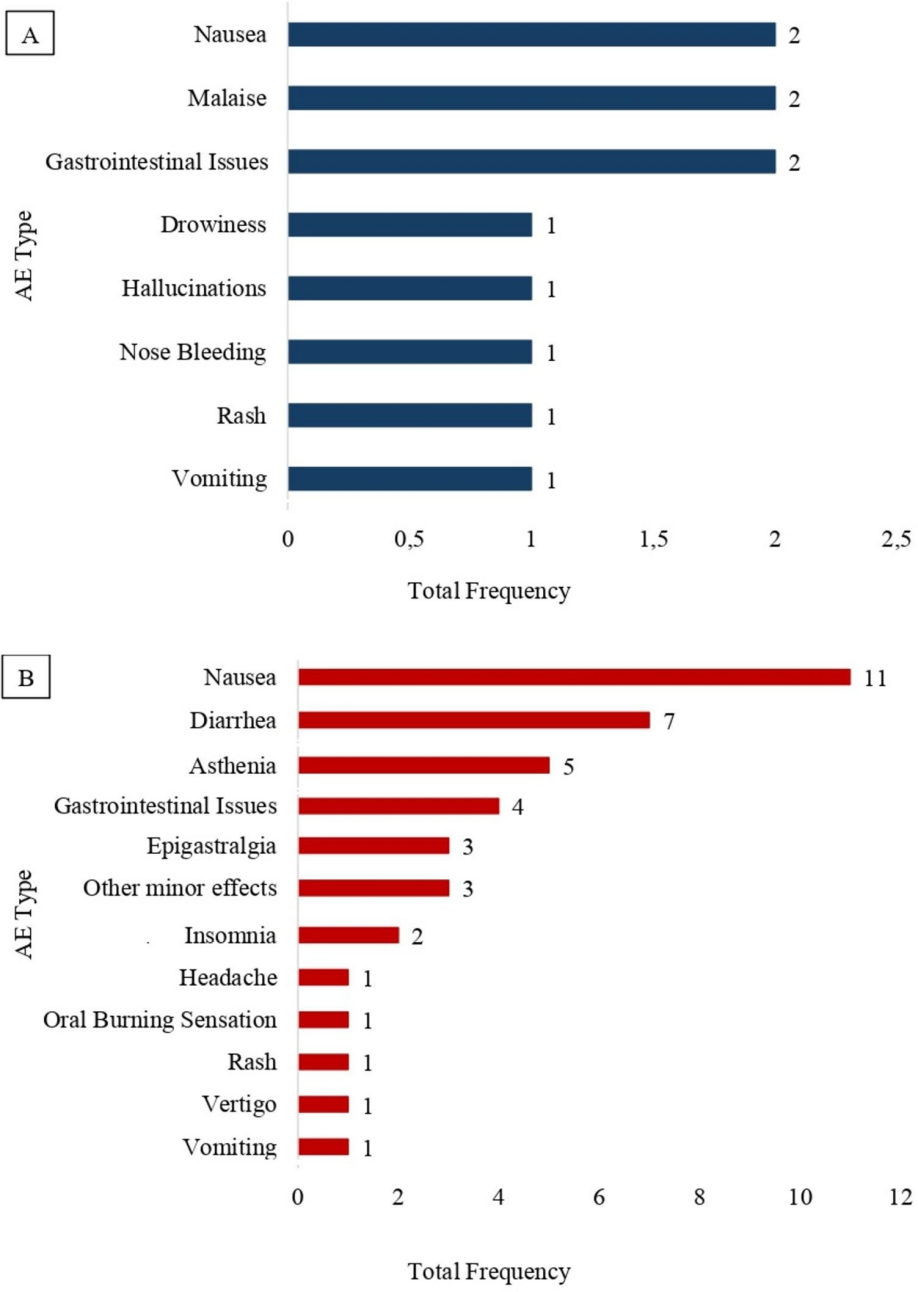
**A)** Total frequency of AEs after PENTO treatment/prevention; **B)** Total frequency of AEs after PENTOCLO treatment

Nausea was the most frequently reported AE, with 13 occurrences (18.84%). Diarrhea accounted for 7 events (10.15%), followed by nonspecific gastrointestinal symptoms, with 6 cases (8.7%). Asthenia was reported in 5 cases (7.25%). Epigastric pain and other minor effects were observed in 3 cases each (4.35%). Malaise, insomnia, vomiting, and rash were each reported in 2 cases (2.9%). Drowsiness, hallucinations, headache, dizziness, nasal bleeding, and oral burning sensation were reported in 1 case each (1.45%). An additional 18 events (26.1%) were not individually specified. When grouped by symptom origin, gastrointestinal symptoms accounted for 32 events (46.38%), neurovegetative symptoms for 12 (17.39%), cutaneous manifestations for 2 (2.9%), and hemorrhagic events for 1 (1.45%). Figures 2A and 2B illustrate the number of AEs associated with the use of PENTO(CLO).

#### Overall pooled estimate

**Fig. 3:**
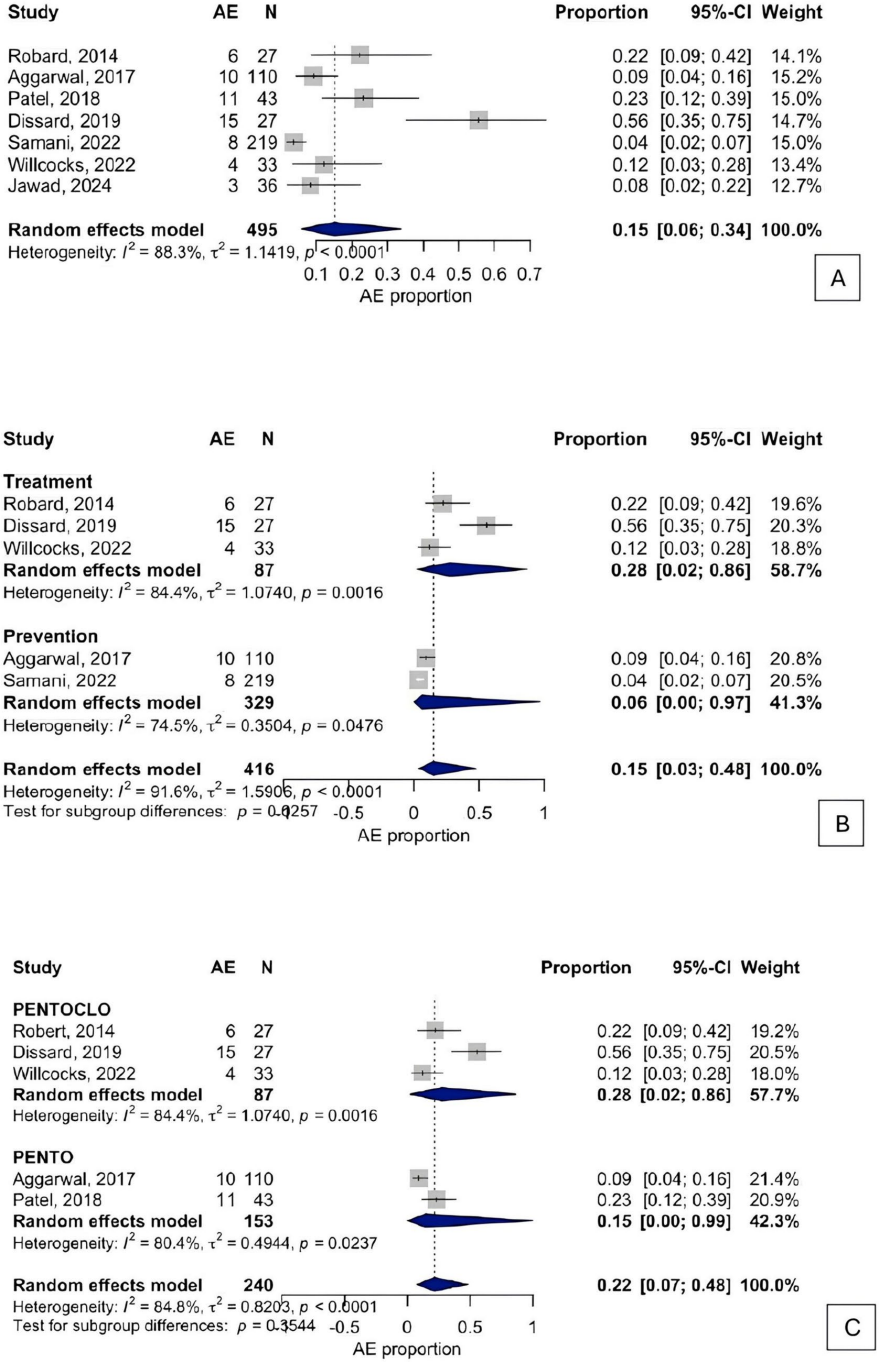
**A)** Overall proportion of adverse effects occurrence; **B)** Proportion of AEs in treatment vs prevention studies; **C)** Proportion of AEs of PENTO vs PENTOCLO

The overall pooled AEs rate was 15% (95% CI: 0.06 to 0.34), with substantial heterogeneity (τ^2^ = 1.1419, I^2^ = 88.3%, 95% CI: 71.0% to 95%; H = 2.92% CI: 1.96 to 4.36) (Fig. 3A). Cochran’s Q test confirm significant heterogeneity (Q = 51.28, df = 6, *p* < 0.0001) (Fig. 3A).

#### Treatment vs Prevention

Five studies (*n* = 416; 43 events) were included in a subgroup analysis exploring PENTOCLO used for treatment (*n* = 3) and prevention (*n* = 2). AEs in treatment was 28% (95% CI: 0% to 97%; I^2^ = 74.5%), and prevention subgroup was 6% (95% CI: 0.00% to 96.72%; I^2^ = 74.5%) **(**Fig. 3B**).** The test for subgroup differences was statistically significant (Q = 5.00, df = 1, *p* = 0.0225), suggesting a higher frequency of AEs in treatment contexts (Fig. 3B).

#### PENTOCLO vs PENTO regimens

PENTOCLO group (*n* = 3) showed a AEs proportion of 28% (95% CI: 2% to 86%; I^2^ = 84.4%), while PENTO group (*n* = 2) resulted in 15% (95% CI: 0% to 99%; I^2^ = 80.4%). The differences between groups were not statistically significant (Q = 4.10, df = 1, *p* = 0.3544) (Fig. 3C).

## Discussion

The administration of PENTO(CLO) protocols has shown promise as an effective alternative for the prevention and management of ORN and MRONJ. This review aimed to identify and synthesize the AEs associated with these regimens and to evaluate their safety profile.

Overall, a relatively low incidence of AEs was observed, approximately 15%, although a wide range was reported across studies, from 4 to 56%, reflecting considerable heterogeneity in the available data. Gastrointestinal disturbances were the most frequently reported events (46.38%), followed by neurovegetative symptoms (18.84%), with cutaneous reactions and hemorrhagic events being less common observed. The most recurrent symptoms were nausea and diarrhea, predominantly occurring within the first four weeks of use, which corresponded to the initial adaptation phase to the protocol. After this period, reports of AEs became infrequent [25, 39, 41].

Relevant differences in AE frequency were noted between the two regimens. While studies involving PENTO reported rates close to the overall average (15%), those employing PENTOCLO showed higher incidences, around 28%. Despite this discrepancy, the nature of the events remained similar across groups, with consistent reports of nausea, epigastralgia, gastric irritation, vomiting, skin rash, and sleep disturbances. Nasal bleeding and malaise were also reported with PENTO. While the addition of clodronate to the PENTOCLO regimen was associated with the occurrence of diarrhea, asthenia, and vertigo, it was also linked to other mild and nonspecific manifestations.

The biological plausibility of the observed AEs is supported by the pharmacological profiles of the agents used. Pentoxifylline, is a methylxanthine derivative, that modulates gastric secretion and may cause hypersecretion and mucosal irritation, which could explain symptoms such as nausea, vomiting, and epigastric pain [42]. Its antiplatelet effects may account for mild hemorrhagic manifestations, including a case of nose bleeding reported in one of the publications [36]. Tocopherol, although generally well tolerated, may also contribute to such events due to its mild anticoagulant properties, which have rarely been associated with intracranial hemorrhage, as described by Owen & Dewald [43]. Skin rash may result from peripheral vasodilation or immunomodulatory mechanisms. Vertigo, headache, and insomnia may be associated with centrally mediated vasodilation induced by pentoxifylline [42, 44].

To improve tolerability, Delanian et al*.* recommend reducing the initial pentoxifylline dose from 800 mg/day to 400 mg/day without compromising therapeutic efficacy [25]. Additionally, some authors suggested that the administration of proton pump inhibitors, such as omeprazole 20 mg twice daily, has shown good results in controlling gastrointestinal symptoms [25, 37]. Regarding diarrhea, Muratore et al*.* suggest that it may result from the irritant action of clodronate on the gastrointestinal mucosa, a mechanism similar to that described for other bisphosphonates in a meta-analysis by Tadrous et al*.* [45, 46]. To minimize this effect, initiating treatment with lower doses, such as 800 mg/day instead of 1600 mg/day, is recommended [25, 33, 37].

Another important aspect is the concomitant use of other substances during protocol administration. Several studies reported protocol associations with antibiotics, corticosteroids, and chlorhexidine [25, 33, 35, 37–39]. The antibiotics used included amoxicillin (with or without clavulanate), clindamycin, ciprofloxacin, penicillin, metronidazole, and doxycycline, all of which have gastrointestinal irritant potential, potentially amplifying the protocol’s AEs, particularly in patients with a history of digestive disorders [47]. Headaches, although multifactorial, have also been associated with the use of ciprofloxacin and amoxicillin-clavulanate. Corticosteroids may induce gastric effects, insomnia, nose bleeding, and neuropsychiatric symptoms such as hallucinations, complicating the accurate attribution of certain AEs to the protocol [48]. Chlorhexidine has a well-documented mucosal irritant effect and was associated with a burning sensation in one study, which resolved completely after its discontinuation [39, 49].

The incidence of AEs also varied depending on the intended use of the protocols. In preventive studies using PENTO alone, the AE rate was approximately 6%. In contrast, therapeutic approaches employing PENTOCLO reported rates around 23%. These findings suggest that patients undergoing active treatment may be more susceptible to AEs. Moreover, all studies involving PENTOCLO were conducted exclusively in therapeutic contexts, which may help explain the higher event rates.

Despite the reported events, the protocol was well tolerated in most cases. Only 15 patients (2.67%) discontinued treatment due to AEs [36, 38, 39]. Patel et al*.* reported six discontinuations, and Samani et al*.* reported eight, all occurring in the early stages of therapy [36, 38]. In the study by Willcocks et al*.*, a single discontinuation was reported, related to persistent gastrointestinal symptoms unresponsive to supportive measures [39].

Although gastrointestinal and neurovegetative symptoms were the most prevalent, cardiovascular, hepatic, and renal AEs must not be overlooked, particularly in high-risk individuals. Jawad et al*.* identified contraindications to pentoxifylline in patients with acute myocardial infarction, cerebral or retinal hemorrhages, and severe arrhythmias, due to its vasodilatory and antiplatelet effects [40]. Additional contraindications include acute porphyria, coronary artery disease, and hypotension. Lombardi et al*.* further cited hypersensitivity to methylxanthines and pregnancy as contraindications [21]. Cardiovascular AE rates ranged from 0.3% to 2.3%, low in absolute terms but potentially serious if unrecognized [50].

Regarding hepatic and renal function, pentoxifylline is generally well tolerated, although transient elevations in liver enzymes have been reported [50]. Since approximately 90% of the drug is excreted renally as water-soluble metabolites, dose adjustment is necessary in patients with creatinine clearance below 30 mL/min to avoid accumulation and toxicity. Similarly, clodronate is excreted unchanged via the kidneys and requires caution in patients with renal impairment [51]. Tocopherol, by contrast, has shown a favorable safety profile at doses up to 1000 IU/day, without reports of serious AEs [44].

Although this review provides a comprehensive synthesis of current evidence, its findings should be interpreted with caution due to several limitations. Most of the included studies were observational and retrospective in nature, carrying a moderate to high risk of bias. The considerable heterogeneity in therapeutic regimens, combined with inconsistencies in AE reporting and management, compromises the precision and comparability of the results. Moreover, AE were often reported only in nonspecific or aggregate terms, with little or no standardized grading, highlighting the need for better-designed clinical studies with systemic AE classification and reporting. Furthermore, the safety profile of these protocols in patients with MRONJ remains insufficiently characterized, and there is a lack of long-term data regarding cardiovascular, hepatic, and renal outcomes across different clinical scenarios. Despite these limitations, the findings offer a valuable foundation for clinical practice and support the cautious application of these protocols.

In summary, this systematic review indicates that PENTO(CLO) protocols show promise as therapeutic options for the prevention and management of ORN and MRONJ, with a relatively low incidence of AEs, most of which were mild and occurred early during treatment. Gastrointestinal and neurovegetative symptoms were the most frequently reported, and higher AE rates were observed with PENTOCLO, likely due to the addition of clodronate and its associated effects. The use of concomitant medications and individual patient conditions may also contribute to the observed AE profiles. Although these protocols were generally well tolerated, particularly when managed with caution, safety concerns persist in more vulnerable populations. There is an urgent need for well-designed and standardized prospective studies to better establish their safety and tolerability.

## Conclusion

Current evidence suggests that the PENTO(CLO) protocol appears to be safe for long-term use, with a low incidence AEs (15%), primarily gastrointestinal and self-limiting. While further studies are needed to elucidate long-term cardiovascular, hepatic, and renal safety, the available evidence support the protocol’s overall tolerability in both preventive and therapeutic contexts.

## Supplementary Information

Below is the link to the electronic supplementary material.

Supplementary file1 (DOCX 20 KB)

Supplementary file2 (DOCX 19 KB)

Supplementary file3 (DOCX 31 KB)

Supplementary file4 (DOCX 14 KB)

Supplementary file5 (JPG 408 KB)

Supplementary file6 (JPG 301 KB)

Supplementary file7 (JPG 342 KB)

Supplementary file8 (DOCX 18 KB)

Supplementary file9 (DOCX 26 KB)

Supplementary file10 (DOCX 37 KB)

## Data Availability

All data supporting the conclusions of this study have been presented in the main text or Supplementary archives.
