# Supplementary material for: Adverse effects of the PENTO(CLO) protocol in the prevention and management of iatrogenic head and neck bone necrosis in cancer patients: A systematic review and meta-analysis
Source: Support Care Cancer. 2026 Feb 20;34(3):224. doi: 10.1007/s00520-026-10428-0 (PMC12920728; doi:10.1007/s00520-026-10428-0)
Supplement: Supplementary file 2 — Supplementary file2 (DOCX 19 KB) [file 520_2026_10428_MOESM2_ESM.docx]

**Search strategy**

| **Database** | **Search strategy**  (Search date: February 24^,^ 2025) | **Results** |
| --- | --- | --- |
| PubMed | (“Pentoxifylline and Tocopherol” OR Pentoxifylline[MeSH Terms] “pentoxifylline-tocopherol-clodronate”[MeSH Terms] OR “clodronic acid”[MeSH Terms] OR PENTOCLO OR pentoxifylline OR oxpentifylline OR trental OR pentoxil OR torental OR agapurin OR “BL-191” OR “BL 191” OR BL191 OR Tocopherols[MeSH Terms] OR tocopherol OR tocovital OR UnoVit OR “Vitamin E” OR vitazell OR detulin OR “E-ferol” OR “E-mulsin” OR “E mulsin” OR “E-Vicotrat” OR “E Vicotrat” OR ecoro OR embial OR evion OR ephynal OR eplonat OR Eusovit OR “Dal-E” OR “Dal E” OR Abortosan OR “Aquasol E” OR “Bio E” OR Biosan OR Lasar OR Bioweyxin OR Davitamon OR Dermorelle OR Spondyvit OR Tocolion OR Tocopa OR Tocopharm OR Vibolex OR “Vita-E” OR “Vita E” OR VitaE OR “Unique E” OR PENTO) **AND** (“Bisphosphonate Associated Osteonecrosis of the Jaw”[MeSH Terms] OR “BRONJ” OR “Osteonecrosis of the Jaws, Bisphosphonate Associated” OR “Bisphosphonate Induced Osteonecrosis of the Jaw” OR “Bisphosphonate Related Osteonecrosis of the Jaw” OR “Osteonecrosis of the Jaw, Bisphosphonate Induced” OR “Osteonecrosis of the Jaw, Bisphosphonate Related” OR “Osteonecrosis of the Jaw, Bisphosphonate Associated” OR “Bisphosphonate Associated Osteonecrosis of the Jaws” OR “Bisphosphonate Associated Osteonecrosis” OR “Osteonecrosis, Bisphosphonate-Associated” OR “Bisphosphonate Osteonecrosis” OR “Osteonecrosis, Bisphosphonate” OR “Osteonecrosis of the Jaw” OR “ONJ” OR “Antiresorptive Agent Related Osteonecrosis of the Jaw” OR “ARONJ” OR “Denosumab-related osteonecrosis of the jaw” OR DRONJ OR “Medication Related Osteonecrosis of the Jaw” OR “MRONJ” OR Osteoradionecrosis[MeSH Terms] OR Osteoradionecroses OR “radiation osteonecrosis”) **AND** (“adverse effects” OR “side effects” OR toxicity OR complications OR “oncological safety”) | 109 |
| Scopus | TITLE-ABS-KEY (“Pentoxifylline and Tocopherol” OR Pentoxifylline “pentoxifylline-tocopherol-clodronate” OR “clodronic acid” OR PENTOCLO OR pentoxifylline OR oxpentifylline OR trental OR pentoxil OR torental OR agapurin OR “BL-191” OR “BL 191” OR BL191 OR Tocopherols OR tocopherol OR tocovital OR UnoVit OR “Vitamin E” OR vitazell OR detulin OR “E-ferol” OR “E-mulsin” OR “E mulsin” OR “E-Vicotrat” OR “E Vicotrat” OR ecoro OR embial OR evion OR ephynal OR eplonat OR Eusovit OR “Dal-E” OR “Dal E” OR Abortosan OR “Aquasol E” OR “Bio E” OR Biosan OR Lasar OR Bioweyxin OR Davitamon OR Dermorelle OR Spondyvit OR Tocolion OR Tocopa OR Tocopharm OR Vibolex OR “Vita-E” OR “Vita E” OR VitaE OR “Unique E” OR PENTO) **AND** TITLE-ABS-KEY (“Bisphosphonate Associated Osteonecrosis of the Jaw” OR “BRONJ” OR “Osteonecrosis of the Jaws, Bisphosphonate Associated” OR “Bisphosphonate Induced Osteonecrosis of the Jaw” OR “Bisphosphonate Related Osteonecrosis of the Jaw” OR “Osteonecrosis of the Jaw, Bisphosphonate Induced” OR “Osteonecrosis of the Jaw, Bisphosphonate Related” OR “Osteonecrosis of the Jaw, Bisphosphonate Associated” OR “Bisphosphonate Associated Osteonecrosis of the Jaws” OR “Bisphosphonate Associated Osteonecrosis” OR “Osteonecrosis, Bisphosphonate-Associated” OR “Bisphosphonate Osteonecrosis” OR “Osteonecrosis, Bisphosphonate” OR “Osteonecrosis of the Jaw” OR “ONJ” OR “Antiresorptive Agent Related Osteonecrosis of the Jaw” OR “ARONJ” OR “Denosumab-related osteonecrosis of the jaw” OR DRONJ OR “Medication Related Osteonecrosis of the Jaw” OR “MRONJ” OR Osteoradionecrosis OR Osteoradionecroses OR “radiation osteonecrosis”) **AND** TITLE-ABS-KEY (“adverse effects” OR “side effects” OR toxicity OR complications OR “oncological safety”) | 104 |
| Embase | ('pentoxifylline and tocopherol' OR 'pentoxifylline' OR 'pentoxifylline'/exp OR pentoxifylline OR 'pentoxifylline-tocopherol-clodronate' OR 'clodronic acid'/exp OR 'clodronic acid' OR pentoclo OR 'oxpentifylline' OR 'oxpentifylline'/exp OR oxpentifylline OR 'trental' OR 'trental'/exp OR trental OR 'pentoxil' OR 'pentoxil'/exp OR pentoxil OR 'torental' OR 'torental'/exp OR torental OR 'agapurin' OR 'agapurin'/exp OR agapurin OR 'bl-191'/exp OR 'bl-191' OR 'bl 191'/exp OR 'bl 191' OR 'bl191' OR 'bl191'/exp OR bl191 OR 'tocopherols' OR 'tocopherols'/exp OR tocopherols OR 'tocopherol' OR 'tocopherol'/exp OR tocopherol OR 'tocovital' OR 'tocovital'/exp OR tocovital OR unovit OR 'vitamin e'/exp OR 'vitamin e' OR vitazell OR 'detulin' OR 'detulin'/exp OR detulin OR 'e-ferol'/exp OR 'e-ferol' OR 'e-mulsin' OR 'e mulsin' OR 'e-vicotrat'/exp OR 'e-vicotrat' OR 'e vicotrat'/exp OR 'e vicotrat' OR ecoro OR embial OR 'evion' OR 'evion'/exp OR evion OR 'ephynal' OR 'ephynal'/exp OR ephynal OR 'eplonat' OR 'eplonat'/exp OR eplonat OR eusovit OR 'dal-e' OR 'dal e' OR abortosan OR 'aquasol e'/exp OR 'aquasol e' OR 'bio e' OR biosan OR lasar OR bioweyxin OR davitamon OR 'dermorelle' OR 'dermorelle'/exp OR dermorelle OR 'spondyvit' OR 'spondyvit'/exp OR spondyvit OR tocolion OR tocopa OR tocopharm OR vibolex OR 'vita-e' OR 'vita e' OR vitae OR 'unique e' OR pento) **AND** ('bisphosphonate associated osteonecrosis of the jaw'/exp OR 'bisphosphonate associated osteonecrosis of the jaw' OR 'bronj'/exp OR bronj OR 'osteonecrosis of the jaws, bisphosphonate associated' OR 'bisphosphonate induced osteonecrosis of the jaw'/exp OR 'bisphosphonate induced osteonecrosis of the jaw' OR 'bisphosphonate related osteonecrosis of the jaw'/exp OR 'bisphosphonate related osteonecrosis of the jaw' OR 'osteonecrosis of the jaw, bisphosphonate induced' OR 'osteonecrosis of the jaw, bisphosphonate related' OR 'osteonecrosis of the jaw, bisphosphonate associated' OR 'bisphosphonate associated osteonecrosis of the jaws' OR 'bisphosphonate associated osteonecrosis' OR 'osteonecrosis, bisphosphonate-associated' OR 'bisphosphonate osteonecrosis' OR 'osteonecrosis, bisphosphonate' OR 'osteonecrosis of the jaw'/exp OR 'osteonecrosis of the jaw' OR onj OR 'antiresorptive agent related osteonecrosis of the jaw'/exp OR 'antiresorptive agent related osteonecrosis of the jaw' OR 'aronj'/exp OR aronj OR 'denosumab-related osteonecrosis of the jaw'/exp OR 'denosumab-related osteonecrosis of the jaw' OR 'dronj'/exp OR dronj OR 'medication related osteonecrosis of the jaw'/exp OR 'medication related osteonecrosis of the jaw' OR 'mronj'/exp OR mronj OR 'osteoradionecrosis'/exp OR osteoradionecrosis OR osteoradionecroses OR 'radiation osteonecrosis'/exp OR 'radiation osteonecrosis') **AND** ('adverse effects'/exp OR 'adverse effects' OR 'side effects' OR 'toxicity'/exp OR toxicity OR 'complications'/exp OR complications OR 'oncological safety') | 494 |
| Web of Science | TS=(“Pentoxifylline and Tocopherol” OR Pentoxifylline “pentoxifylline-tocopherol-clodronate” OR “clodronic acid” OR PENTOCLO OR pentoxifylline OR oxpentifylline OR trental OR pentoxil OR torental OR agapurin OR “BL-191” OR “BL 191” OR BL191 OR Tocopherols OR tocopherol OR tocovital OR UnoVit OR “Vitamin E” OR vitazell OR detulin OR “E-ferol” OR “E-mulsin” OR “E mulsin” OR “E-Vicotrat” OR “E Vicotrat” OR ecoro OR embial OR evion OR ephynal OR eplonat OR Eusovit OR “Dal-E” OR “Dal E” OR Abortosan OR “Aquasol E” OR “Bio E” OR Biosan OR Lasar OR Bioweyxin OR Davitamon OR Dermorelle OR Spondyvit OR Tocolion OR Tocopa OR Tocopharm OR Vibolex OR “Vita-E” OR “Vita E” OR VitaE OR “Unique E” OR PENTO) **AND** TS=(“Bisphosphonate Associated Osteonecrosis of the Jaw” OR “BRONJ” OR “Osteonecrosis of the Jaws, Bisphosphonate Associated” OR “Bisphosphonate Induced Osteonecrosis of the Jaw” OR “Bisphosphonate Related Osteonecrosis of the Jaw” OR “Osteonecrosis of the Jaw, Bisphosphonate Induced” OR “Osteonecrosis of the Jaw, Bisphosphonate Related” OR “Osteonecrosis of the Jaw, Bisphosphonate Associated” OR “Bisphosphonate Associated Osteonecrosis of the Jaws” OR “Bisphosphonate Associated Osteonecrosis” OR “Osteonecrosis, Bisphosphonate-Associated” OR “Bisphosphonate Osteonecrosis” OR “Osteonecrosis, Bisphosphonate” OR “Osteonecrosis of the Jaw” OR “ONJ” OR “Antiresorptive Agent Related Osteonecrosis of the Jaw” OR “ARONJ” OR “Denosumab-related osteonecrosis of the jaw” OR DRONJ OR “Medication Related Osteonecrosis of the Jaw” OR “MRONJ” OR Osteoradionecrosis OR Osteoradionecroses OR “radiation osteonecrosis”) **AND** TS=(“adverse effects” OR “side effects” OR toxicity OR complications OR “oncological safety”) | 69 |
| LILACS | (Pentoxifilina OR Pentoxifylline OR Tocoferol OR Tocoferoles OR “Ácido Clodrônico” OR “Clodronic Acid” OR “Acide clodronique”) AND (“Osteonecrose da Arcada Osseodentária Associada a Difosfonatos” OR “Osteonecrose Associada a Bifosfonatos” OR “Osteonecrose Associada a Difosfonatos” OR “Osteonecrose Associada aos Bisfosfonatos” OR “Osteonecrose Associada aos Difosfonatos” OR “Osteonecrose da Arcada Osseodentária Associada a Bisfosfonatos” OR “Osteonecrose da Arcada Osseodentária Associada aos Bisfosfonatos” OR “Osteonecrose da Arcada Osseodentária por Bisfosfonatos” OR “Osteonecrose da Arcada Osseodentária por Difosfonatos” OR “Bisphosphonate Associated Osteonecrosis” OR “Bisphosphonate Associated Osteonecrosis of the Jaw” OR “Bisphosphonate Associated Osteonecrosis of the Jaws” OR “Bisphosphonate Induced Osteonecrosis of the Jaw” OR “Bisphosphonate Induced Osteonecrosis of the Jaws” OR “Ostéonécrose de la mâchoire associée aux biphosphonates” OR “Osteorradionecrose” OR “Osteorradionecrosis” OR “Osteoradionecrosis” OR “Ostéoradionécrose”) AND (“Efeitos Adversos” OR “Efeito Colateral e Reação Adversa Relacionados a Medicamentos” OR “Efeitos Colaterais e Reações Adversas Associados a Medicamentos” OR “Evento Adverso” OR “Eventos Adversos” OR “Reações Adversas e Efeitos Colaterais Relacionados a Drogas” OR “Reações Adversas e Efeitos Colaterais Relacionados a Medicamentos” OR “Drug-Related Side Effects and Adverse Reactions” OR “Efectos Colaterales y Reacciones Adversas Relacionados con Medicamentos” OR “Effets secondaires indésirables des médicaments”) | 32 |
| Cochrane Library | (“Pentoxifylline and Tocopherol” OR Pentoxifylline “pentoxifylline-tocopherol-clodronate” OR “clodronic acid” OR PENTOCLO OR pentoxifylline OR oxpentifylline OR trental OR pentoxil OR torental OR agapurin OR “BL-191” OR “BL 191” OR BL191 OR Tocopherols OR tocopherol OR tocovital OR UnoVit OR “Vitamin E” OR vitazell OR detulin OR “E-ferol” OR “E-mulsin” OR “E mulsin” OR “E-Vicotrat” OR “E Vicotrat” OR ecoro OR embial OR evion OR ephynal OR eplonat OR Eusovit OR “Dal-E” OR “Dal E” OR Abortosan OR “Aquasol E” OR “Bio E” OR Biosan OR Lasar OR Bioweyxin OR Davitamon OR Dermorelle OR Spondyvit OR Tocolion OR Tocopa OR Tocopharm OR Vibolex OR “Vita-E” OR “Vita E” OR VitaE OR “Unique E” OR PENTO) **AND** (“Bisphosphonate Associated Osteonecrosis of the Jaw” OR “BRONJ” OR “Osteonecrosis of the Jaws, Bisphosphonate Associated” OR “Bisphosphonate Induced Osteonecrosis of the Jaw” OR “Bisphosphonate Related Osteonecrosis of the Jaw” OR “Osteonecrosis of the Jaw, Bisphosphonate Induced” OR “Osteonecrosis of the Jaw, Bisphosphonate Related” OR “Osteonecrosis of the Jaw, Bisphosphonate Associated” OR “Bisphosphonate Associated Osteonecrosis of the Jaws” OR “Bisphosphonate Associated Osteonecrosis” OR “Osteonecrosis, Bisphosphonate-Associated” OR “Bisphosphonate Osteonecrosis” OR “Osteonecrosis, Bisphosphonate” OR “Osteonecrosis of the Jaw” OR “ONJ” OR “Antiresorptive Agent Related Osteonecrosis of the Jaw” OR “ARONJ” OR “Denosumab-related osteonecrosis of the jaw” OR DRONJ OR “Medication Related Osteonecrosis of the Jaw” OR “MRONJ” OR Osteoradionecrosis OR Osteoradionecroses OR “radiation osteonecrosis”) **AND** (“adverse effects” OR “side effects” OR toxicity OR complications OR “oncological safety”) | 23 |
| **Grey Literature** | | |
| Google Scholar | First 100 more relevant hits. No patents and no citations.  (pentoxifylline OR tocoferol OR clodronate OR clodronic acid OR PENTO OR PENTOCLO) AND ("Bisphosphonate-related osteonecrosis of the jaw" OR "Medication-related osteonecrosis of the jaw" OR "Desnosumab-related osteonecrosis of the jaw" OR BRONJ OR MRONJ OR DRONJ OR osteoradionecrosis OR osteorradionecrose OR ORN) AND (“adverse effects” OR “side effects” OR toxicity OR complications OR “oncological safety”) | 100 |
| ProQuest | TI,AB(“Pentoxifylline and Tocopherol” OR Pentoxifylline “pentoxifylline-tocopherol-clodronate” OR “clodronic acid” OR PENTOCLO OR pentoxifylline OR oxpentifylline OR trental OR pentoxil OR torental OR agapurin OR “BL-191” OR “BL 191” OR BL191 OR Tocopherols OR tocopherol OR tocovital OR UnoVit OR “Vitamin E” OR vitazell OR detulin OR “E-ferol” OR “E-mulsin” OR “E mulsin” OR “E-Vicotrat” OR “E Vicotrat” OR ecoro OR embial OR evion OR ephynal OR eplonat OR Eusovit OR “Dal-E” OR “Dal E” OR Abortosan OR “Aquasol E” OR “Bio E” OR Biosan OR Lasar OR Bioweyxin OR Davitamon OR Dermorelle OR Spondyvit OR Tocolion OR Tocopa OR Tocopharm OR Vibolex OR “Vita-E” OR “Vita E” OR VitaE OR “Unique E” OR PENTO) **AND** TI,AB(“Bisphosphonate Associated Osteonecrosis of the Jaw” OR “BRONJ” OR “Osteonecrosis of the Jaws, Bisphosphonate Associated” OR “Bisphosphonate Induced Osteonecrosis of the Jaw” OR “Bisphosphonate Related Osteonecrosis of the Jaw” OR “Osteonecrosis of the Jaw, Bisphosphonate Induced” OR “Osteonecrosis of the Jaw, Bisphosphonate Related” OR “Osteonecrosis of the Jaw, Bisphosphonate Associated” OR “Bisphosphonate Associated Osteonecrosis of the Jaws” OR “Bisphosphonate Associated Osteonecrosis” OR “Osteonecrosis, Bisphosphonate-Associated” OR “Bisphosphonate Osteonecrosis” OR “Osteonecrosis, Bisphosphonate” OR “Osteonecrosis of the Jaw” OR “ONJ” OR “Antiresorptive Agent Related Osteonecrosis of the Jaw” OR “ARONJ” OR “Denosumab-related osteonecrosis of the jaw” OR DRONJ OR “Medication Related Osteonecrosis of the Jaw” OR “MRONJ” OR Osteoradionecrosis OR Osteoradionecroses OR “radiation osteonecrosis”) **AND** (“adverse effects” OR “side effects” OR toxicity OR complications OR “oncological safety”) | 37 |
| ClinicalTrials.gov | (“Pentoxifylline and Tocopherol” OR Pentoxifylline “pentoxifylline-tocopherol-clodronate” OR “clodronic acid” OR PENTOCLO OR pentoxifylline OR oxpentifylline OR trental OR pentoxil OR torental OR agapurin OR “BL-191” OR “BL 191” OR BL191 OR Tocopherols OR tocopherol OR tocovital OR UnoVit OR “Vitamin E” OR vitazell OR detulin OR “E-ferol” OR “E-mulsin” OR “E mulsin” OR “E-Vicotrat” OR “E Vicotrat” OR ecoro OR embial OR evion OR ephynal OR eplonat OR Eusovit OR “Dal-E” OR “Dal E” OR Abortosan OR “Aquasol E” OR “Bio E” OR Biosan OR Lasar OR Bioweyxin OR Davitamon OR Dermorelle OR Spondyvit OR Tocolion OR Tocopa OR Tocopharm OR Vibolex OR “Vita-E” OR “Vita E” OR VitaE OR “Unique E” OR PENTO) AND (“Bisphosphonate Associated Osteonecrosis of the Jaw” OR “BRONJ” OR “Osteonecrosis of the Jaws, Bisphosphonate Associated” OR “Bisphosphonate Induced Osteonecrosis of the Jaw” OR “Bisphosphonate Related Osteonecrosis of the Jaw” OR “Osteonecrosis of the Jaw, Bisphosphonate Induced” OR “Osteonecrosis of the Jaw, Bisphosphonate Related” OR “Osteonecrosis of the Jaw, Bisphosphonate Associated” OR “Bisphosphonate Associated Osteonecrosis of the Jaws” OR “Bisphosphonate Associated Osteonecrosis” OR “Osteonecrosis, Bisphosphonate-Associated” OR “Bisphosphonate Osteonecrosis” OR “Osteonecrosis, Bisphosphonate” OR “Osteonecrosis of the Jaw” OR “ONJ” OR “Antiresorptive Agent Related Osteonecrosis of the Jaw” OR “ARONJ” OR “Denosumab-related osteonecrosis of the jaw” OR DRONJ OR “Medication Related Osteonecrosis of the Jaw” OR “MRONJ” OR Osteoradionecrosis OR Osteoradionecroses OR “radiation osteonecrosis”) AND (“adverse effects” OR “side effects” OR toxicity OR complications OR “oncological safety”) | 7 |
| Ovid | (“Pentoxifylline and Tocopherol” OR Pentoxifylline “pentoxifylline-tocopherol-clodronate” OR “clodronic acid” OR PENTOCLO OR pentoxifylline OR oxpentifylline OR trental OR pentoxil OR torental OR agapurin OR “BL-191” OR “BL 191” OR BL191 OR Tocopherols OR tocopherol OR tocovital OR UnoVit OR “Vitamin E” OR vitazell OR detulin OR “E-ferol” OR “E-mulsin” OR “E mulsin” OR “E-Vicotrat” OR “E Vicotrat” OR ecoro OR embial OR evion OR ephynal OR eplonat OR Eusovit OR “Dal-E” OR “Dal E” OR Abortosan OR “Aquasol E” OR “Bio E” OR Biosan OR Lasar OR Bioweyxin OR Davitamon OR Dermorelle OR Spondyvit OR Tocolion OR Tocopa OR Tocopharm OR Vibolex OR “Vita-E” OR “Vita E” OR VitaE OR “Unique E” OR PENTO) AND (“Bisphosphonate Associated Osteonecrosis of the Jaw” OR “BRONJ” OR “Osteonecrosis of the Jaws, Bisphosphonate Associated” OR “Bisphosphonate Induced Osteonecrosis of the Jaw” OR “Bisphosphonate Related Osteonecrosis of the Jaw” OR “Osteonecrosis of the Jaw, Bisphosphonate Induced” OR “Osteonecrosis of the Jaw, Bisphosphonate Related” OR “Osteonecrosis of the Jaw, Bisphosphonate Associated” OR “Bisphosphonate Associated Osteonecrosis of the Jaws” OR “Bisphosphonate Associated Osteonecrosis” OR “Osteonecrosis, Bisphosphonate-Associated” OR “Bisphosphonate Osteonecrosis” OR “Osteonecrosis, Bisphosphonate” OR “Osteonecrosis of the Jaw” OR “ONJ” OR “Antiresorptive Agent Related Osteonecrosis of the Jaw” OR “ARONJ” OR “Denosumab-related osteonecrosis of the jaw” OR DRONJ OR “Medication Related Osteonecrosis of the Jaw” OR “MRONJ” OR Osteoradionecrosis OR Osteoradionecroses OR “radiation osteonecrosis”) AND (“adverse effects” OR “side effects” OR toxicity OR complications OR “oncological safety”) | 100 |

* It was not possible to perform the search on Open Grey as the website did not provide a search option.
