# Supplementary material for: Adverse effects of the PENTO(CLO) protocol in the prevention and management of iatrogenic head and neck bone necrosis in cancer patients: A systematic review and meta-analysis
Source: Support Care Cancer. 2026 Feb 20;34(3):224. doi: 10.1007/s00520-026-10428-0 (PMC12920728; doi:10.1007/s00520-026-10428-0)
Supplement: Supplementary file 3 — Supplementary file3 (DOCX 31 KB) [file 520_2026_10428_MOESM3_ESM.docx]

| **Author/Year** | **Source** | **Title** | **Reason for Study Exclusion** | **Available** |
| --- | --- | --- | --- | --- |
| Martos-Fernandez, 2018 | Grey Literature | Management of Mandibular ORN: PENTO as Medical Treatment (PENTO) | Insufficient data available | https://clinicaltrials.gov/study/NCT02368457 |
| Dillon, 2017 | Grey Literature | Pentoxifylline and Tocopherol (PENTO) in the Treatment of Medication-related Osteonecrosis of the Jaw (MRONJ) | Insufficient data available | https://clinicaltrials.gov/study/NCT03040778 |
| Usseglio, 2023 | Grey Literature | The PENTO Protocol in Medication-related Osteonecrosis of the Jaw (PENTO) | Insufficient data available | https://clinicaltrials.gov/study/NCT05795647 |
| Safa, 2023 | Grey Literature | Combination Therapy: Hyperbaric Oxygen and PENTOCLO for Treatment of Osteoradionecrosis of the Mandible | Insufficient data available | https://clinicaltrials.gov/study/NCT06055257 |
| AlHakim *et al.*, 2024 | Grey Literature | The management of osteoradionecrosis in palliative head and neck cancer patients | Wrong Publication Type | https://onlinelibrary.wiley.com/doi/full/10.1111/ors.12867 |
| Bulsara *et al.*, 2019 | Grey Literature | Protocol for prospective randomised assessor-blinded pilot study comparing hyperbaric oxygen therapy with PENtoxifylline+TOcopherol± CLOdronate for the management of early osteoradionecrosis of the mandible | Wrong Outcome | https://www.proquest.com/docview/2187743175?accountid=8113&sourcetype=Scholarly%20Journals |
| Colapinto *et al.*, 2023 | Grey Literature | Outcomes of a Pharmacological Protocol with Pentoxifylline and Tocopherol for the Management of Medication-Related Osteonecrosis of the Jaws (MRONJ): A Randomized Study on 202 Osteoporosis Patients | Wrong Outcome | https://doi.org/10.3390/jcm12144662 |
| Farajollah *et al.,* 2024 | Grey Literature | The Impact Of Purine Derivatives Therapy For Patients With Medication-Related Osteonecrosis Of The Jaw: Preliminary Results From A Pilot Study – The Sequel | Wrong Outcome | https://rjor.ro/the-impact-of-purine-derivatives-therapy-for-patients-with-medication-related-osteonecrosis-of-the-jaw-preliminary-results-from-a-pilot-study-the-sequel/ |
| Feitosa *et al.*, 2022 | Grey Literature | Prevention Of Osteoradionecrosis Of The Jaws With The Combination Of Pentoxifylline And Tocopherol | Wrong Publication Type | https://www.sciencedirect.com/science/article/pii/S2212440322007349 |
| Patel *et al.,* 2021 | Grey Literature | The use of pentoxifylline, tocopherol and clodronate in the management of osteoradionecrosis of the jaws | Wrong Outcome | https://www.thegreenjournal.com/article/S0167-8140(20)31253-6/fulltext |
| Słowik *et al.,* 2025 | Grey Literature | Pharmacological Treatment of Medication-Related Osteonecrosis of the Jaw (MRONJ) with Pentoxifylline and Tocopherol | Wrong Outcome | <https://doi.org/10.3390/jcm14030974> |
| Delanian 2005 | Data Base | Major healing of refractory mandible osteoradionecrosis after treatment combining pentoxifylline and tocopherol: A phase II trial[^†^](https://onlinelibrary.wiley.com/doi/10.1002/hed.20121#fn1) | Wrong Outcome | https://doi.org/10.1002/hed.20121 |
| D’Souza 2014 | Data Base | Changing trends and the role of medical management on the outcome of patients treated for osteoradionecrosis of the mandible: experience from a regional head and neck unit | Wrong Outcome | https://doi.org/10.1016/j.bjoms.2014.01.003 |
| Patel 2015 | Data Base | The Prophylactic Use of Pentoxifylline and Tocopherol to Avoid Osteoradionecrosis for Dental Implant Placement in Irradiated Head and Neck Cancer Patients | Wrong Publication Type | https://doi.org/10.1016/j.joms.2015.06.049 |
| Kulkarni *et al.*, 2015 | Data Base | The role of Pentoxifylline–Tocopherol–Clodronate (PENTOCLO) in osteoradionecrosis (ORN) of the mandible | Wrong Publication Type | https://doi.org/10.1016/j.bjoms.2015.08.078 |
| Patel *et al.*, 2016 | Data Base | Use of pentoxifylline and tocopherol in the management of osteoradionecrosis | Wrong Outcome | https://doi.org/10.1016/j.bjoms.2015.11.027 |
| Ghazali *et al.*, 2016 | Data Base | Evaluating Outcomes of Combination Pentoxifylline and Tocopherol in Preventing Osteradionecrosis of the Jaws | Wrong Publication Type | https://doi.org/10.1016/j.joms.2016.06.072 |
| Martos-Fernandez, 2016 | Data Base | Management of Mandibular ORN: PENTO as Medical Treatment (PENTO) | Insufficient data available | https://clinicaltrials.gov/study/NCT02368457 |
| Owosho *et al.*, 2016 | Data Base | Pentoxifylline and tocopherol in the management of cancer patients with medication-related osteonecrosis of the jaw: an observational retrospective study of initial case series | Wrong publication type | https://doi.org/10.1016/j.oooo.2016.06.019 |
| Dillon, 2017 | Data Base | Pentoxifylline and Tocopherol (PENTO) in the Treatment of Medication-related Osteonecrosis of the Jaw (MRONJ) | Insufficient data available | https://clinicaltrials.gov/study/NCT03040778 |
| Bulsara, 2018 | Data Base | Protocol for prospective randomised assessor-blinded pilot study comparing hyperbaric oxygen therapy with PENtoxifylline+TOcopherol± CLOdronate for the management of early osteoradionecrosis of the mandible | Wrong Outcome | https://doi.org/10.1136/bmjopen-2018-026662 |
| Patel *et al.*, 2019 | Data Base | A clinic feasibility study to assess whether the use of two combined medicines (pentoxifylline and tocopherol) can prevent radiotherapy-related changes of the mouth and face compared to the current standard of care in the head and neck cancer population | Insufficient data available | https://doi.org/10.1186/ISRCTN74484952 |
| Patel *et al.*, 2020 | Data Base | The use of pentoxifylline, tocopherol and clodronate in the management of osteoradionecrosis of the jaws | Wrong Outcome | https://doi.org/10.1016/j.radonc.2020.12.027 |
| Rawcliffe, 2022 | Data Base | Clinical trial of the non-surgical management of radiotherapy damage to the lower jaw | Insufficient data available | https://doi.org/10.1186/ISRCTN34217298 |
| Lombardi, 2022 | Data Base | Pentoxifylline and tocopherol for prevention of osteoradionecrosis in patients who underwent oral surgery: A clinical audit | Wrong Outcome | https://doi.org/10.1111/scd.12759 |
| Colapinto *et al.*, 2023 | Data Base | Outcomes of a Pharmacological Protocol with Pentoxifylline and Tocopherol for the Management of Medication-Related Osteonecrosis of the Jaws (MRONJ): A Randomized Study on 202 Osteoporosis Patients | Wrong Outcome | https://doi.org/10.3390/jcm12144662 |
| Gul *et al.*, 2023 | Data Base | Effectiveness of Pentoxifylline and Tocopherol in patients with Osteoradionecrosis | Wrong Outcome | https://doi.org/10.48047/ |
| Safa, 2023 | Data Base | Combination Therapy: Hyperbaric Oxygen and PENTOCLO for Treatment of Osteoradionecrosis of the Mandible | Insufficient data available | https://clinicaltrials.gov/study/NCT06055257 |
| Farajollah *et al.*, 2024 | Data Base | The impact of purine derivatives therapy for patients with medication-related osteonecrosis of the jaw: preliminary results from a pilot study | Wrong Outcome | https://doi.org/10.62610/rjor.2024.3.16.8 |
| Kanatas *et al.*, 2024 | Data Base | RAPTOR: Randomised Controlled Trial of PENTOCLO in Mandibular Osteoradionecrosis | Wrong Publication Type | https://doi.org/10.1016/j.ejso.2023.107194 |
| Mahendran *et al.*, 2024 | Data Base | Insights and a Retrospective Analysis from a Single Institution using Pentoxifylline, Vitamin E, and Sodium Clodronate in the Prevention and Management of ORN | Wrong Publication Type | https://doi.org/10.1016/j.joms.2024.06.045 |
| Arcoverde, 2024 | Data Base | RBR-6tnqpjm Prevention of Bone Necrosis induced by Radiotherapy in the oral cavity region with the drug combination of Pentoxifyllin | Insufficient data available | https://ensaiosclinicos.gov.br/rg/RBR-6tnqpjm |
| Slowik *et al.*, 2025 | Data Base | Pharmacological Treatment of Medication-Related Osteonecrosis of the Jaw (MRONJ) with Pentoxifylline and Tocopherol | Wrong Outcome | https://doi.org/https://doi.org/10.3390/jcm14030974 |
