# Supplementary material for: Adverse effects of the PENTO(CLO) protocol in the prevention and management of iatrogenic head and neck bone necrosis in cancer patients: A systematic review and meta-analysis
Source: Support Care Cancer. 2026 Feb 20;34(3):224. doi: 10.1007/s00520-026-10428-0 (PMC12920728; doi:10.1007/s00520-026-10428-0)
Supplement: Supplementary file 4 — Supplementary file4 (DOCX 14 KB) [file 520_2026_10428_MOESM4_ESM.docx]

**GLOSSARY**

AEs – Adverse Effects

CI – Confidence Interval

CLO – Clodronate (Clodronic Acid)

MRONJ – Medication-Related Osteonecrosis of the Jaw

ORN – Osteoradionecrosis

PECOS - Population, Exposure, Comparator, Outcome, and Study Design

PEN – Pentoxifylline

PENTO – Pentoxifylline + Tocopherols

PENTOCLO – Pentoxifylline + Tocopherols + Clodronate

PROSPERO - International Prospective Register of Systematic Reviews

RR – Risk Ratio

TO – Tocopherols
