# Supplementary figures and images for: Adverse effects of the PENTO(CLO) protocol in the prevention and management of iatrogenic head and neck bone necrosis in cancer patients: A systematic review and meta-analysis
Source: Support Care Cancer. 2026 Feb 20;34(3):224. doi: 10.1007/s00520-026-10428-0 (PMC12920728; doi:10.1007/s00520-026-10428-0)

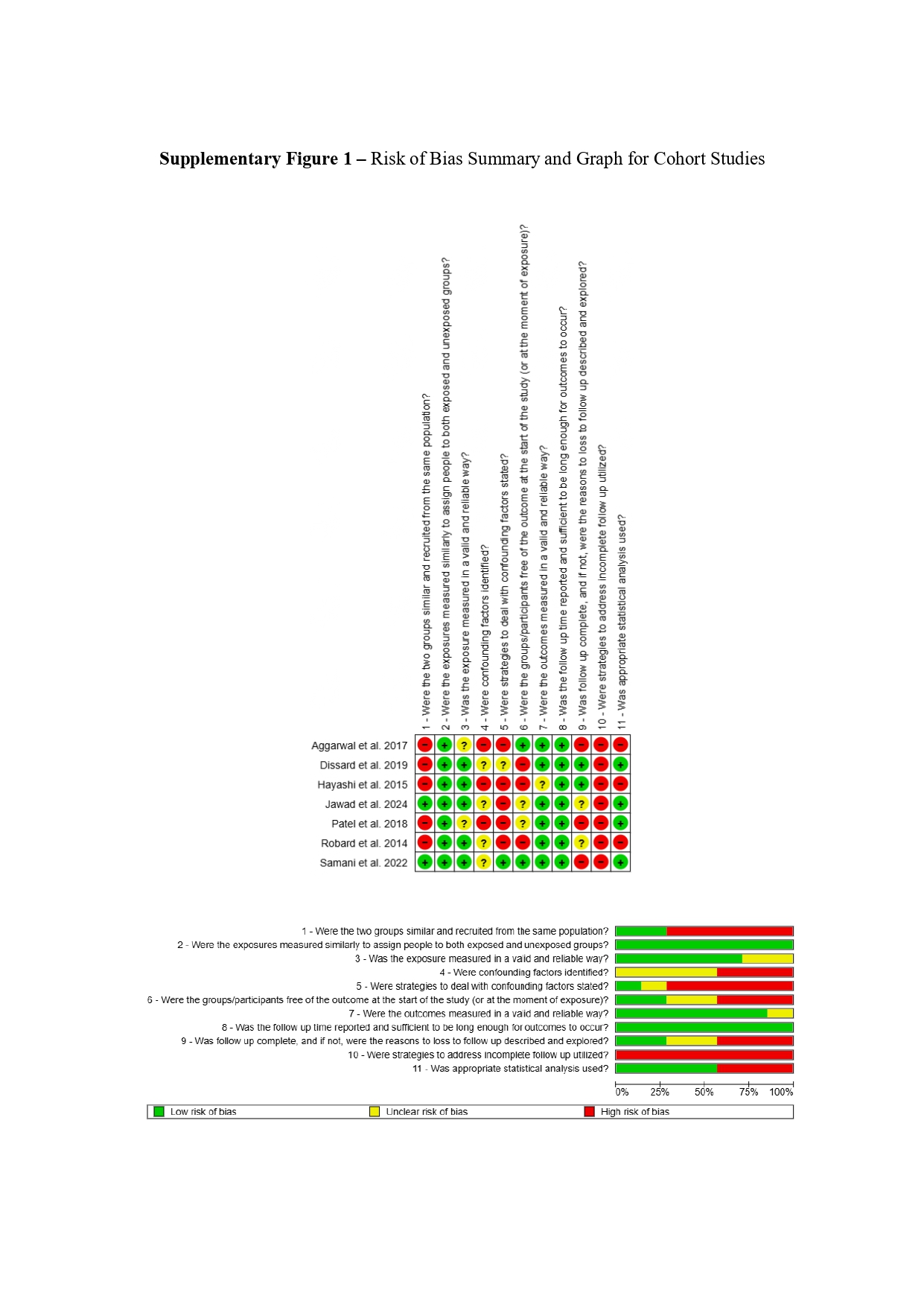

Supplement: Supplementary file 5 — Supplementary file5 (JPG 408 KB) [file 520_2026_10428_MOESM5_ESM.jpg]

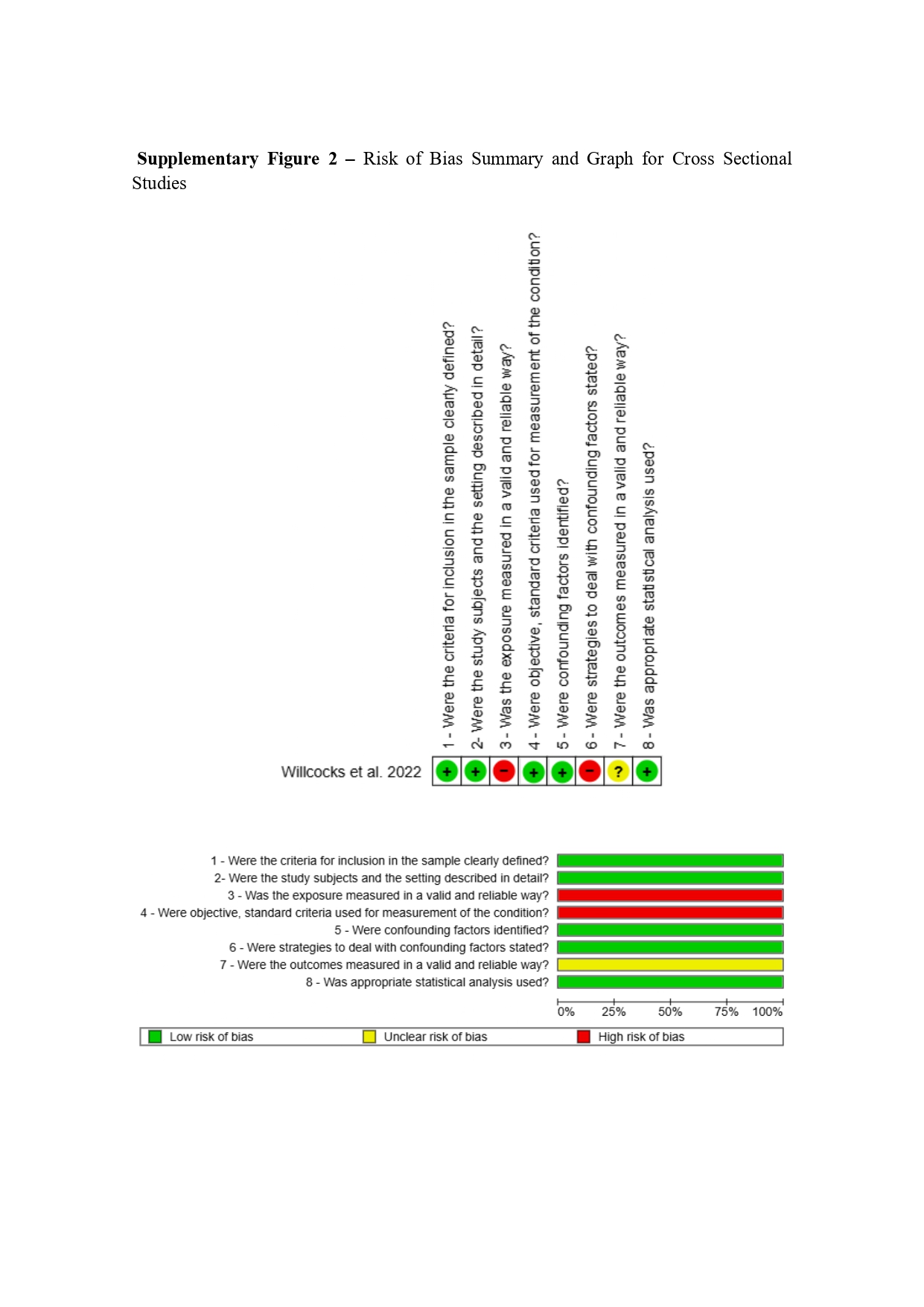

Supplement: Supplementary file 6 — Supplementary file6 (JPG 301 KB) [file 520_2026_10428_MOESM6_ESM.jpg]

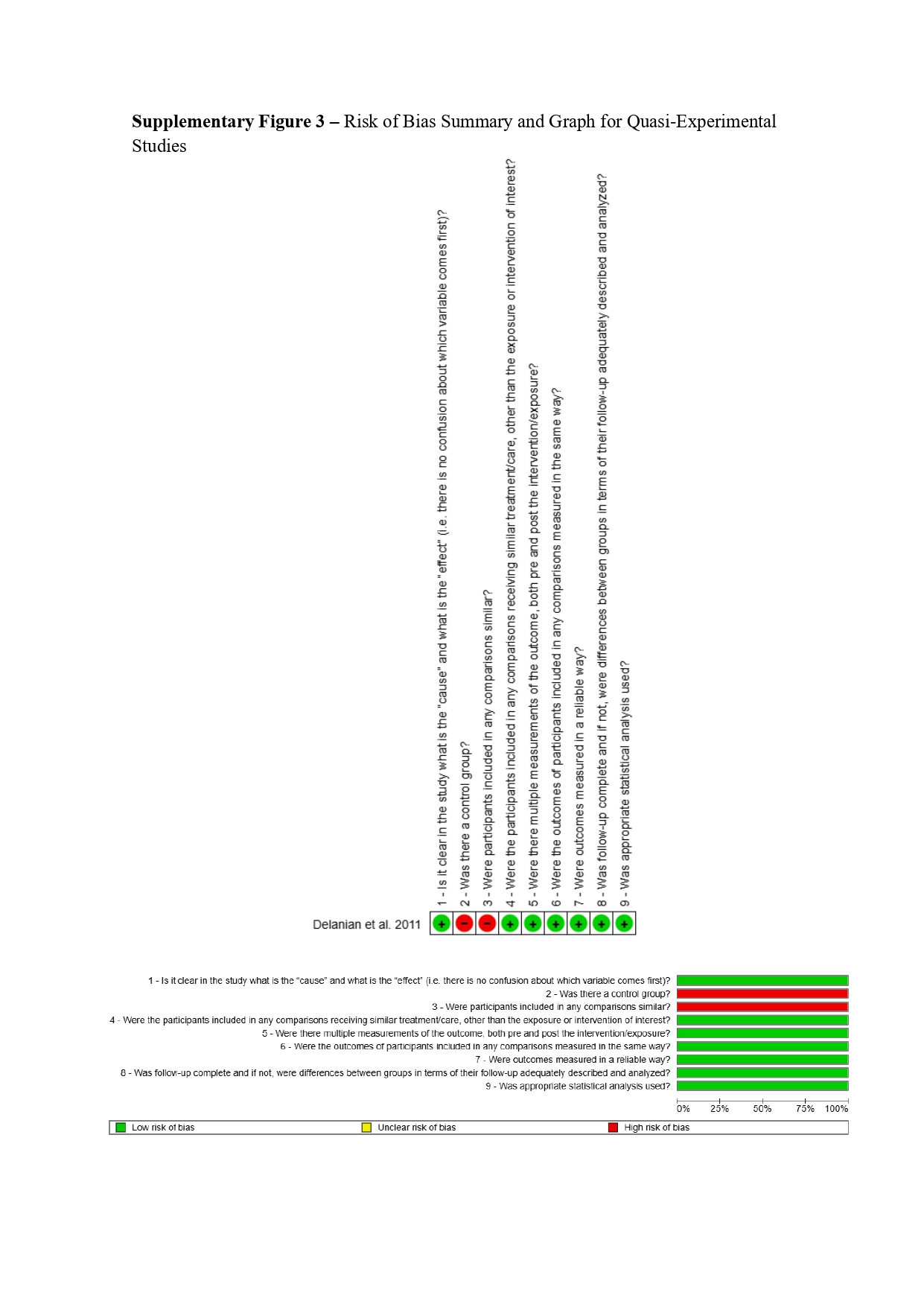

Supplement: Supplementary file 7 — Supplementary file7 (JPG 342 KB) [file 520_2026_10428_MOESM7_ESM.jpg]
