# Supplementary material for: Adverse effects of the PENTO(CLO) protocol in the prevention and management of iatrogenic head and neck bone necrosis in cancer patients: A systematic review and meta-analysis
Source: Support Care Cancer. 2026 Feb 20;34(3):224. doi: 10.1007/s00520-026-10428-0 (PMC12920728; doi:10.1007/s00520-026-10428-0)
Supplement: Supplementary file 8 — Supplementary file8 (DOCX 18 KB) [file 520_2026_10428_MOESM8_ESM.docx]

| *Author/ Year* | | *Country* | | *Study design* | | *Objective* | |
| --- | --- | --- | --- | --- | --- | --- | --- |
|  |  |  |  |  |  |  | |
| Delanian *et al.,* 2011 | | France | | Non-Randomized Clinical Trial | | Treatment | |
| Robard *et al., 2014* | | France | | Retrospective Cohort | | Treatment | |
| Hayashi *et al.*, 2015 | | United States | | Retrospective Cohort | | Treatment | |
| Aggarwal *et al.*, 2017 | | India | | Retrospective Cohort | | Prevention | |
| Patel *et al.*, 2018 | | United Kingdom | | Retrospective Cohort | | Prevention/Treatment | |
| Dissard *et al.*, 2019 | | France | | Prospective Cohort | | Treatment | |
| Samani *et al.*, 2022 | | United Kingdom | | Retrospective Cohort | | Prevention | |
| Willcocks *et al.*, 2022 | | United Kingdom | | Cross-Sectional | | Treatment | |
| Jawad *et al.*, 2024 | | United Kingdom | | Retrospective Cohort | | Prevention/Treatment | |

**Supplementary Table 1** - Characteristics of the included studies
