# Supplementary material for: Adverse effects of the PENTO(CLO) protocol in the prevention and management of iatrogenic head and neck bone necrosis in cancer patients: A systematic review and meta-analysis
Source: Support Care Cancer. 2026 Feb 20;34(3):224. doi: 10.1007/s00520-026-10428-0 (PMC12920728; doi:10.1007/s00520-026-10428-0)
Supplement: Supplementary file 9 — Supplementary file9 (DOCX 26 KB) [file 520_2026_10428_MOESM9_ESM.docx]

| **Author/ Year** | **Study Design** | **Sample - Total** (n) | **Treatment or Prevention** | **Protocol PENTO/PENTOCLO** | **Protocol Regimen** | **Protocol Supportive Medications** | | | **Protocol Duration (months)** | | |  |
| --- | --- | --- | --- | --- | --- | --- | --- | --- | --- | --- | --- | --- |
|  |  |  |  |  |  | **Antibiotic** | **Corticosteroids** | **Proton Pump Inhibitors (PPI)** | **Mean** | Min | Max |  |
| Delanian *et al.,* 2011 | Non-Randomized Clinical Trial | 54 | Treatment | PENTOCLO | PTX 800mg+ TCP 1000 IU+ CLO 1600mg - 5 days/week | Ciprofloxacin 1g - 2 days/week | Prednisone 20mg - 2 days/week | Reported (Table 2) | 16 | 6 | 36 |  |
|  |  |  |  |  |  |  |  |  |  |  |  |  |
|  |  |  |  |  |  |  |  |  |  |  |  |  |
|  |  |  |  |  |  |  |  |  |  |  |  |  |
|  |  |  |  |  |  |  |  |  |  |  |  |  |
|  |  |  |  |  |  |  |  |  |  |  |  |  |
|  |  |  |  |  |  |  |  |  |  |  |  |  |
| Robard *et al., 2014* | Retrospective Cohort | 27 | Treatment | PENTOCLO | PTX 800mg+ TCP 1000 IU+ CLO 1600mg - 5 days/week | Not Reported | Prednisone 20mg - 2 days/week | Not Reported | 3 | 1 | 8 |  |
|  |  |  |  |  |  |  |  |  |  |  |  |  |
| Hayashi *et al.*, 2015 | Retrospective Cohort | 13 | Treatment | PENTO | PTX 800mg+TCP 1000 IU - daily | Not Reported | Not Reported | Not Reported | 13,5 | 1 | 33 |  |
| Aggarwal *et al.*, 2017 | Retrospective Cohort | 110 | Prevention | PENTO | PTX 800mg+TCP 1000 IU - daily | Penicilin or Penicilin + Metronidazole | Not Reported | Not Reported | 6,5 | Not Reported | Not Reported |  |
|  |  |  |  |  |  |  |  |  |  |  |  |  |
|  |  |  |  |  |  |  |  |  |  |  |  |  |
| Patel *et al.*, 2018 | Retrospective Cohort | 43 | Treatment / Prevention | PENTO | Lacking sufficient clarity | Not Reported | Not Reported | Not Reported | Not Reported | Not Reported | Not Reported |  |
|  |  |  |  |  |  |  |  |  |  |  |  |  |
|  |  |  |  |  |  |  |  |  |  |  |  |  |
|  |  |  |  |  |  |  |  |  |  |  |  |  |
|  |  |  |  |  |  |  |  |  |  |  |  |  |
|  |  |  |  |  |  |  |  |  |  |  |  |  |
|  |  |  |  |  |  |  |  |  |  |  |  |  |
|  |  |  |  |  |  |  |  |  |  |  |  |  |
| Dissard *et al.*, 2019 | Prospective Cohort | 27 | Treatment | PENTOCLO | PTX 800mg+ TCP 1000 IU+ CLO 1600mg - 5 days/week | Not Reported | Prednisone 20mg - 2 days/week | Omeprazole 20mg - 2 days/week | 9,3 | 1 | 24 |  |
|  |  |  |  |  |  |  |  |  |  |  |  |  |
|  |  |  |  |  |  |  |  |  |  |  |  |  |
|  |  |  |  |  |  |  |  |  |  |  |  |  |
|  |  |  |  |  |  |  |  |  |  |  |  |  |
| Samani *et al.*, 2022 | Retrospective Cohort | 219 | Prevention | PENTO OR PENTOCLO | PTX 800mg+TCP 1000 IU/ daily or PTX 800mg+TCP 1000 IU + CLO 1600mg/ daily | Amoxicillin 500mg - 7 first days or Metronidazole 400mg- 7 first days or Clindamycin 300mg- 7 first days | Not Reported | Not Reported | Not Reported | 1 | 37 |  |
| Willcocks *et al.*, 2022 | Cross-Sectional | 33 | Treatment | PENTOCLO | PTX +TCP + DOX+CHX or PTX+TCP+CLO or PTX+TCP+CLO+DOX* | Doxycycline* | Not Reported | Not Reported | Not Reported | Not Reported | Not Reported |  |
|  |  |  |  |  |  |  |  |  |  |  |  |  |
|  |  |  |  |  |  |  |  |  |  |  |  |  |
|  |  |  |  |  |  |  |  |  |  |  |  |  |
|  |  |  |  |  |  |  |  |  |  |  |  |  |
| Jawad *et al.*, 2024 | Retrospective Cohort | 36 | Treatment/ Prevention | PENTO OR PENTOCLO | PTX 800mg+TCP 1000 IU/ daily or PTX 800mg+TCP 1000 IU + CLO 1600mg/ daily | Not Reported | Not Reported | Not Reported | 4,7-16,7 | Not Reported | Not Reported |  |

PTX: Pentoxifylline; TCP: Tocopherol; CLO: Clodronate; DOX: Doxycycline

**Supplementary Table 2.** General and Specific Characteristics of the Included Studies
