# Supplementary material for: Adverse effects of the PENTO(CLO) protocol in the prevention and management of iatrogenic head and neck bone necrosis in cancer patients: A systematic review and meta-analysis
Source: Support Care Cancer. 2026 Feb 20;34(3):224. doi: 10.1007/s00520-026-10428-0 (PMC12920728; doi:10.1007/s00520-026-10428-0)
Supplement: Supplementary file 10 — Supplementary file10 (DOCX 37 KB) [file 520_2026_10428_MOESM10_ESM.docx]

**Supplementary Table 3.1.** Risk of Bias Summary of Cohort Studies According to Reviewers’ Judgments Using the Joanna Briggs Institute Critical Appraisal Checklist

| Nº | Author | Year | 1. | 2. | 3. | 4. | 5. | | 6. | 7. | 8. | 9. | 10. | 11. | % | Risk |
| --- | --- | --- | --- | --- | --- | --- | --- | --- | --- | --- | --- | --- | --- | --- | --- | --- |
| 1 | Robard *et al.* | 2014 | N | Y | Y | Unc | N | N | | Y | Y | Unc | N | N | 45,5 | High |
| 2 | Hayashi *et al.* | 2015 | N | Y | Y | N | N | N | | Unc | Y | Y | N | N | 40,9 | High |
| 3 | Aggarwal *et al.* | 2017 | N | Y | Unc | N | N | Y | | Y | Y | N | N | N | 40,9 | High |
| 4 | Patel *et al.* | 2018 | N | Y | Unc | N | N | Unc | | Y | Y | N | N | Y | 45,5 | High |
| 5 | Dissard *et al.* | 2019 | N | Y | Y | Unc | Unc | N | | Y | Y | Y | N | Y | 63,7 | Moderate |
| 6 | Samani *et al.* | 2022 | Y | Y | Y | Y | Y | Y | | Y | Y | N | N | Y | 81,8 | Low |
| 7 | Jawad *et al.* | 2024 | Y | Y | Y | Unc | N | Unc | | Y | Y | Unc | N | Y | 68,2 | Moderate |

| 1. Were the two groups similar and recruited from the same population? |
| --- |
| 1. Were the exposures measured similarly to assign people to both exposed and unexposed groups? |
| 1. Was the exposure measured in a valid and reliable way? |
| 1. Were confounding factors identified? |
| 1. Were strategies to deal with confounding factors stated? |
| 1. Were the groups/participants free of the outcome at the start of the study (or at the moment of exposure)? |
| 1. Were the outcomes measured in a valid and reliable way? |
| 1. Was the follow up time reported and sufficient to be long enough for outcomes to occur? |
| 1. Was follow up complete, and if not, were the reasons to loss to follow up described and explored? |
| 1. Were strategies to address incomplete follow up utilized? |
| 1. Was appropriate statistical analysis used? |

**Supplementary Table 3.2.** Risk of Bias Summary for Cross-Sectional Studies According to Reviewers’ Judgments Using the Joanna Briggs Institute Critical Appraisal Checklist

| Nº | Author | Year | 1. | 2. | 3. | 4. | 5. | 6. | 7. | 8. | % | Risk |
| --- | --- | --- | --- | --- | --- | --- | --- | --- | --- | --- | --- | --- |
| 1 | Willcocks *et al.* | 2022 | Y | Y | N | Y | Y | N | Unc | Y | 68,75 | Moderate |

| 1. Were the criteria for inclusion in the sample clearly defined? |
| --- |
| 1. Were the study subjects and the setting described in detail? |
| 1. Was the exposure measured in a valid and reliable way? |
| 1. Were objective, standard criteria used for measurement of the condition? |
| 1. Were confounding factors identified? |
| 1. Were strategies to deal with confounding factors stated? |
| 1. Were the outcomes measured in a valid and reliable way? |
| 1. Was appropriate statistical analysis used? |
|  |

| Nº | Author | Year | 1. | 2. | 3. | 4. | 5. | 6. | 7. | 8. | 9. | % | Risk |
| --- | --- | --- | --- | --- | --- | --- | --- | --- | --- | --- | --- | --- | --- |
| 1 | Delanian *et al.* | 2011 | Y | N | N | Y | Y | Y | Y | Y | Y | 77,8 | Low |
|  |  |  |  |  |  |  |  |  |  |  |  |  |  |

**Supplementary Table 3.3.** Risk of Bias Summary for Quasi-Experimental Studies According to Reviewers’ Judgments Using the Joanna Briggs Institute Critical Appraisal Checklist

| 1. Is it clear in the study what is the “cause” and what is the “effect” (i.e. there is no confusion about which variable comes first)? |
| --- |
| 1. Was there a control group? |
| 1. Were participants included in any comparisons similar? |
| 1. Were the participants included in any comparisons receiving similar treatment/care, other than the exposure or intervention of interest? |
| 1. Were there multiple measurements of the outcome, both pre and post the intervention/exposure? |
| 1. Were the outcomes of participants included in any comparisons measured in the same way? |
| 1. Were outcomes measured in a reliable way? |
| 1. Was follow-up complete and if not, were differences between groups in terms of their follow-up adequately described and analyzed? 2. Was appropriate statistical analysis used? |
|  |
